# It is not the best option to perform transurethral enucleation of the prostate immediately after biopsy in patients with histological inflammation

**DOI:** 10.3389/fsurg.2024.1390656

**Published:** 2024-07-01

**Authors:** Tengfei Gu, Jie Li, Ting Chen, Yongtao Pan, Jing Sha

**Affiliations:** ^1^ Department of Urology, Lishui Municipal Central Hospital, The Fifth Affiliated Hospital of Wenzhou Medical University, Lishui, China; ^2^ Department of Nursing, Lishui Municipal Central Hospital, The Fifth Affiliated Hospital of Wenzhou Medical University, Lishui, China

**Keywords:** prostatic hyperplasia, prostatic histological inflammation, bipolar enucleation of the prostate, clinical efficacy, prostate biopsy, prospective research

## Abstract

**Objective:**

This study seeks to investigate the impact of histopathological evidence of histological prostatic inflammation (PI) on the surgical outcomes of patients with benign prostatic hyperplasia (BPH) undergoing transurethral bipolar enucleation of the prostate (BiLEP) after biopsy.

**Methods:**

We conducted a prospective study in which data were collected from 112 patients with BPH who underwent BiLEP immediately after prostate biopsy at the Department of Urology in our hospital between October 2020 and October 2023. This cohort included 52 patients with histopathological prostatic inflammation (BPH + PI group) and 60 patients with simple BPH (BPH group). Baseline characteristics, surgical details, International Prostate Symptom Score (IPSS), quality of life (QoL), post-void residual volume (PVR), maximum flow rate (Qmax), International Index of Erectile Function-5 (IIEF-5), postoperative pathology results, and surgical complications were compared between the two groups.

**Results:**

The study findings indicate that in patients with BPH who underwent BiLEP, various parameters in the BPH + PI group including operation time, intraoperative flushing volume, hemoglobin drop value, postoperative white blood cells, postoperative C-reactive protein, and average pain score at 3 days postoperatively were significantly higher compared to those in the BPH group (*p* < 0.01). In addition, the IPSS and IIEF-5 scores of the BPH + PI group were significantly worse before surgery and at 2 weeks postoperatively compared to the BPH group (*p* < 0.01); however, no significant differences were observed between the two groups at 1 and 3 months postoperatively (*p* > 0.05). At 2 weeks postoperatively, the BPH + PI group exhibited significantly worse outcomes in terms of QoL, PVR, and Qmax compared to the BPH group (*p* < 0.01). However, there were no statistically significant differences between the two groups at 1 and 3 months postoperatively (*p* > 0.05). The incidence rates of postoperative complications, such as fever, prostatic capsule perforation, urinary tract irritation, bladder spasm, acute epididymitis, urinary tract infection, and urethral stricture, were higher in the BPH + PI group compared to the BPH group (*p* < 0.05). Nevertheless, there was no significant difference in the overall complication rates between the two groups (*p* > 0.05). There were no statistically significant differences observed between the two groups in postoperative irrigation volume, extubation time, hospitalization time, proportion of secondary operations, proportion of bladder injury, and proportion of urinary incontinence (*p* > 0.05). However, the proportion of reported prostate cancer after surgery in the BPH + PI group was significantly higher than that in the BPH group (*p* < 0.05).

**Conclusion:**

Histopathological prostatic inflammation does not have a significant impact on the long-term efficacy of BiLEP surgery immediately after biopsy. However, it does prolong surgery time, increase surgery-related complications, and influence short-term surgical outcomes and patient treatment experience. Therefore, it may be advisable to administer a course of anti-inflammatory treatment before performing BiLEP in such patients. Nevertheless, further high-quality studies are necessary to validate this approach.

## Introduction

Benign prostatic hyperplasia (BPH) is a prevalent condition among elderly men, with an overall incidence rate of 50% and a prevalence rate of approximately 70% in men aged over 70 years (1). Prostatic inflammation (PI), another common urinary disease in men, has an incidence rate of about 13% in the population (2), with a significantly higher occurrence among patients with prostatic hyperplasia. Research indicates that approximately 20% of individuals with prostatic hyperplasia experience complications related to prostatic histological inflammation (3). Furthermore, studies have demonstrated that 78% of individuals with prostatic hyperplasia exhibit inflammatory cell infiltration in postoperative pathology (4). While prostate-specific antigen (PSA) is a crucial serum marker for diagnosing prostate cancer, numerous studies have highlighted that prostatic hyperplasia can also lead to elevated levels of PSA in the bloodstream (5, 6). In addition, as the condition advances, the levels of PSA tend to rise as well. Research has indicated that patients presenting with prostatic hyperplasia and prostatic histological inflammation may experience elevated levels of PSA (7), necessitating prostate biopsy before treatment initiation.

Bipolar enucleation of the prostate (BiLEP) is the primary surgical approach for managing prostatic hyperplasia, and studies have shown that different enucleation methods have similar effects (8). Nevertheless, the presence of concomitant prostatic histological inflammation in patients with prostatic hyperplasia can complicate the surgical procedure. Several studies have indicated that patients with prostatic histological inflammation, as opposed to those with simple prostatic hyperplasia, may experience inferior surgical outcomes and increased surgical complications (9). However, it is important to note that these findings are based on retrospective analyses of postoperative pathological results. In patients presenting with concurrent surgical and biopsy indications for BPH, a preoperative biopsy is necessary so that prostate histological inflammation is often detected preoperatively. Limited research exists on the potential impact of prostate inflammation on prostate enucleation immediately after biopsy. This study conducted a follow-up on patients who underwent transurethral plasma enucleation of the prostate immediately after prostate biopsy to investigate the impact of prostatic histological inflammation on the procedure and to determine the optimal treatment approach for these patients.

## Materials and methods

### Participants

**Figure 1:**
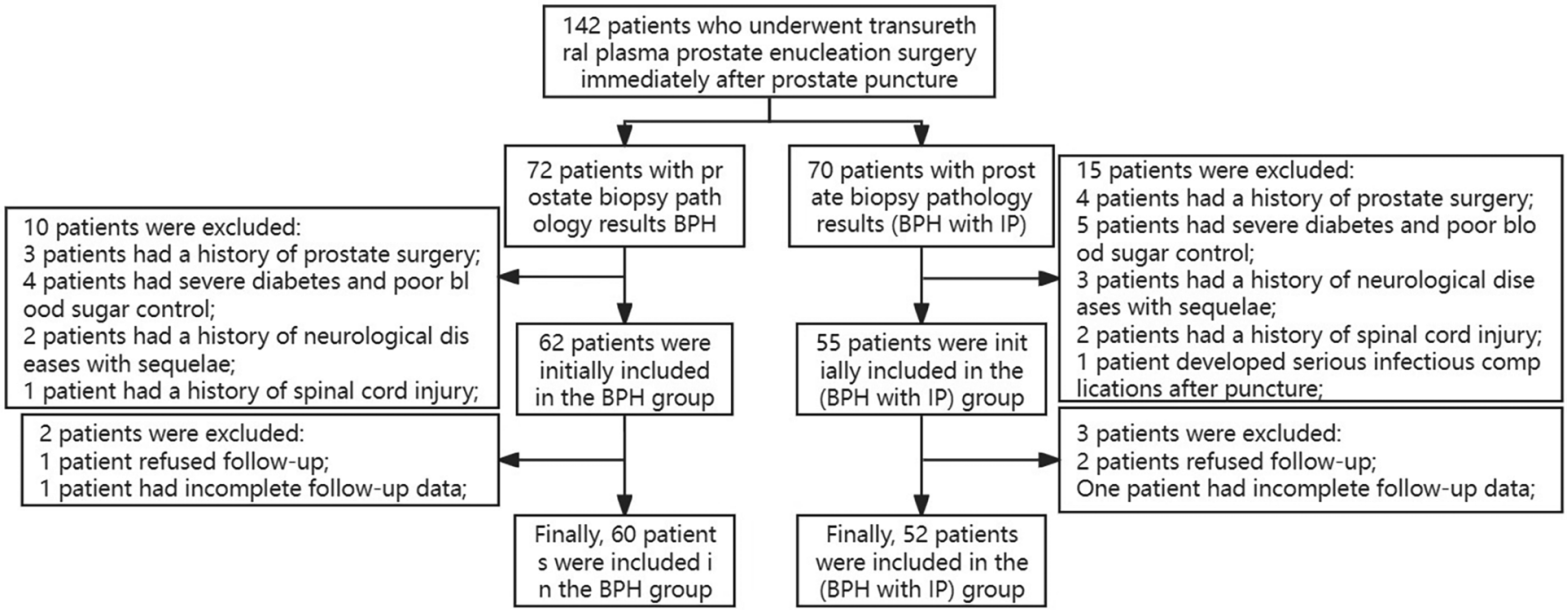
Flowchart of patients in this study.

This study received approval from the Ethics Committee of Lishui Central Hospital in Zhejiang, China, and utilized a prospective design. The study enrolled 142 patients with prostatic hyperplasia who underwent transurethral plasma prostate enucleation surgery after prostate biopsy between October 2020 and October 2023. The inclusion criteria encompassed patients with biopsy-confirmed prostatic hyperplasia or prostatic hyperplasia with concomitant prostatitis, presenting clinical symptoms of prostatic hyperplasia that were unresponsive to conservative treatment, and who were aged older than 55 years. The exclusion criteria comprised patients with a prior history of prostate surgery, post-biopsy antibiotic use, spinal injury or other neurological disorders, severe comorbidities, and BPH surgery conducted more than 3 weeks after biopsy. A total of 10 cases were excluded from the BPH group and 15 from the BPH + PI group based on these criteria. In addition, during the 3-month postoperative follow-up, two patients from the BPH group and three from the BPH + PI group were excluded due to incomplete follow-up. A total of 112 cases were included in the study, with 60 cases in the BPH group and 52 cases in the BPH + PI group. The patient admission and discharge flow chart is depicted in Figure 1.

### Grouping

According to the prostate biopsy results, participants with or without prostatic histological inflammation were categorized into the BPH + PI group or the BPH group, respectively.

### Methods

A total of 112 patients diagnosed with prostatic hyperplasia and elevated PSA levels underwent transperineal prostate biopsy. After the biopsy pathology results, the patients were categorized into two groups: those with BPH + PI and those with pure BPH. It is imperative to actively manage the comorbidities present in the patients, such as hypertension, diabetes, and coronary heart disease, before surgery and ensure they are under control before proceeding with the surgical intervention. Patients unable to achieve control of their comorbid conditions within a 3-week timeframe will be excluded from the study. All patients in both cohorts underwent general anesthesia in the lithotomy position, with surgeries performed by two experienced urological surgeons specializing in prostate enucleation procedures. The BiLEP was performed using the F26 resectoscope of Gyrus plasmakinetic system (Gyrus Medical, England, UK) with a cutting power of 140–160 W and a coagulating power of 80–100 W. Tissue morcellation was achieved using the HAWK Great White Shark medical surgical planer, with 0.9% saline solution utilized for irrigation during the BiLEP procedure. The surgical procedure utilizes the three-lobe technique, beginning with excision of the middle lobe followed by excision of the left and right lateral lobes. The excised tissue is then pushed into the bladder then adequate hemostasis. Subsequently, a tissue breaker is utilized to crush and extract the prostate tissue. An F20 Foley catheter is left in place after the operation. The catheter should be flushed routinely after surgery, and the catheter should be removed when the hematuria stops and the outflow is completely clear.

### Study variables

The patient's preoperative data (including age, time from surgery to prostate biopsy, prostate volume, comorbidities, and serum PSA) were collected from medical records and perioperative data (including operation time, irrigation volume and time, hemoglobin drop value, postoperative inflammation indicators, postoperative pain score, indwelling urinary catheter time, and hospital stay) were obtained and recorded. Complications or adverse events (including secondary postoperative hemorrhage, blood transfusion, bladder spasm, urinary tract irritation, prostatic capsule perforation, bladder injury, and urethral stricture) were obtained and recorded perioperatively or during follow-up and referred to Clavien–Dindo classification classifies each complication according to levels I–V. Hemoglobin, white blood cells, and C-reactive protein levels were detected within 24 h after surgery, and urinary tract infection was diagnosed through urine culture. The International Prostate Symptom Score (IPSS), Quality of Life Score (QoL), maximum urinary flow rate (Qmax), and residual urine volume (PVR) were assessed before surgery and 2 weeks, 1 month, and 3 months after surgery.

Abdominal ultrasound and transrectal ultrasound were used to measure PVR and prostate volume, respectively, and sexual function was evaluated using the International Index of Erectile Function (IIEF-5). During follow-up, if the patient was missing data for IPSS, QoL, Qmax, PVR and IIEF-5 once, the missing values of the parameters could be filled by imputation or by averaging those of the completed patients. If a patient was missing these parameters more than twice, the patient was considered lost to follow-up.

### Statistical analysis

SPSS Statistics for Windows, version 22.0 (IBM Corp., Armonk, NY, USA) was used to perform the statistical analysis. Normally distributed measurement data were expressed as mean ± standard deviation (*x* ± SD), the independent sample *t*-test was used for comparison between groups, and skewed distribution measurement data were expressed as median (range), using the Mann–Whitney *U* test. Categorical data are expressed as percentages (%) and analyzed using the chi-square test or Fisher's exact test. A *p*-value <0.05 is considered statistically significant.

## Results

### Comparison of baseline data between the two groups

In the analysis of preoperative data, it was observed that the average total PSA (tPSA) level in the BPH + PI group was significantly higher than that in the BPH group (*p* < 0.05). In addition, a greater number of patients in the BPH + PI group required catheterization after biopsy compared to the BPH group (*p* < 0.05). However, no statistically significant variances were noted in other preoperative baseline characteristics between the two groups (*p* > 0.05), as shown in Table 1.

**Table 1:** Comparison of baseline data between the two groups [(*x*¯ ± SD), *n* (%)].

Index	BPH + PI group	BPH group	*t* or *x*^2^	*p*
Age (years)	70.25 ± 5.98	70.48 ± 6.01	1.688	0.09
Time from operation to biopsy (days)	12.04 ± 4.03	12.21 ± 3.87	0.747	0.455
BPH drug treatment time (months)	13.80 ± 4.68	13.91 ± 4.58	0.406	0.685
Prostate volume (ml)	67.71 ± 10.05	67.47 ± 6.83	1.244	0.214
Catheterization before biopsy (*n*)	8 (15.38)	5 (8.3)	1.350	0.245
Catheterization after biopsy (*n*)	16 (30.77)	8 (13.33)	5.030	0.025
Bladder stones (*n*)	11 (21.15)	14 (23.33)	0.076	0.782
Hypertension (*n*)	27 (51.92)	31 (51.67)	0.001	0.978
Diabetes (*n*)	15 (28.85)	19 (31.67)	0.105	0.746
Coronary heart disease (*n*)	14 (26.92)	16 (43.33)	0.001	0.976
Cerebral infarction (*n*)	14 (26.92)	16 (43.33)	0.001	0.976
History of taking antiplatelet	18 (34.62)	24 (40)	0.345	0.557
Drugs (*n*) tPSA (ng/ml)	8.27 ± 2.21	7.88 ± 2.28	2.516	0.012
Urodynia VAS score	2.81 ± 0.89	2.73 ± 0.83	0.845	0.399
ASA classification (I–II)	46 (88.46)	52 (86.67)	0.082	0.775

VAS, visual analog score; ASA, American Society of Anesthesiologists.

### Comparison of perioperative data between the two groups

The comparison of perioperative data between the BPH + PI group and the BPH group revealed that the former exhibited higher values in operation time, intraoperative saline flushing volume, operation-related hemoglobin drop, postoperative white blood cells, postoperative C-reactive protein, and postoperative pain scores within 3 days (*p* < 0.01). However, there were no statistically significant differences in postoperative saline flushing volume, flushing time, extubation time, and discharge time between the two groups (*p* > 0.05) as shown in Table 2.

**Table 2:** Comparison of perioperative data between the two groups (*x*¯ ± SD).

Index	BPH + PI group	BPH group	*t* or *x*^2^	*p*
Operation time (min)	68.07 ± 8.07	57.97 ± 6.88	55.99	<0.001
Intraoperative irrigation volume (L)	18.18 ± 4.21	16.28 ± 3.17	10.94	<0.001
Preoperative hemoglobin (g/L)	120.31 ± 10.13	119.98 ± 11.34	1.783	0.075
Postoperative hemoglobin (g/L)	107.36 ± 8.28	109.84 ± 9.37	15.291	<0.001
Hemoglobin drop value (g/L)	16.22 ± 6.12	12.34 ± 4.94	12.227	<0.001
Postoperative white blood cells (10^9^/L)	11.47 ± 1.55	10.02 ± 1.51	16.26	<0.001
Postoperative C-reactive protein (mg/L)	19.99 ± 6.89	12.23 ± 1.39	29.858	<0.001
Postoperative irrigation days	1.53 ± 0.5	1.55 ± 0.5	0.235	0.815
Postoperative irrigation volume (L)	13.25 ± 3.16	13.09 ± 3.06	0.968	0.333
Time to remove urinary catheter (days)	2.46 ± 0.5	2.48 ± 0.5	0.310	0.757
Postoperative hospital stay (days)	3.59 ± 0.72	3.56 ± 0.59	0.469	0.639
Average value of VAS 3 days after surgery	3.11 ± 0.43	2.24 ± 0.41	17.763	<0.001

### Comparison of IPSS, QoL, Qmax, PVR, and IIEF-5 between the two groups

The BPH + PI group and the BPH group were followed up for 3 months. The IPSS, QoL, Qmax, PVR, and IIEF-5 of the two groups were compared before surgery and 2 weeks, 1 month, and 3 months after surgery. The IPSS score before surgery and 2 weeks after surgery, and the IIEF-5 score before surgery, 2 weeks after surgery, and 1 month after surgery in the BPH + PI group were worse than those in the BPH group (*p* < 0.05), but at 3 months postoperatively, there was no significant difference in IPSS scores and IIEF-5 scores between the two groups (*p* > 0.05). The QoL, Qmax, and PVR at 2 weeks postoperatively in the BPH + PI group were worse than those in the BPH group (*p* < 0.05). However, there were no significant differences in QoL, Qmax, and PVR between the two groups before surgery and 1 month and 3 months after surgery (*p* > 0.05) (Table 3).

**Table 3:** Comparison of IPSS, QoL, Qmax, PVR, and IIEF-5 between the two groups (*x*¯ ± SD).

Group	*N*	Preoperative	2 weeks	1 month	3 months
IPSS					
BPH + PI	52	25.71 ± 1.89	16.42 ± 1.20	10.76 ± 0.97	7.01 ± 0.85
BPH	60	24.75 ± 1.44	14.33 ± 1.57	10.67 ± 1.49	6.94 ± 1.07
*t*		15.286	30.734	1.306	1.001
*p*		<0.001	<0.001	0.192	0.317
QoL					
BPH + PI	52	4.26 ± 0.86	2.34 ± 0.59	1.63 ± 0.63	1.37 ± 0.49
BPH	60	4.14 ± 0.58	1.99 ± 0.79	1.48 ± 0.50	1.46 ± 0.50
*t*		1.885	3.66	1.611	1.035
*p*		0.067	<0.001	0.109	0.302
Qmax (ml/s)					
BPH + PI	52	7.53 ± 1.04	23.08 ± 1.61	25.65 ± 1.15	25.77 ± 1.06
BPH	60	7.46 ± 0.89	24.72 ± 1.81	25.68 ± 1.13	25.82 ± 1.11
*t*		1.018	24.427	0.824	1.002
*p*		0.309	<0.001	0.410	0.316
PVR (ml)					
BPH + PI	52	135.50 ± 23.41	28.92 ± 6.87	18.45 ± 4.31	15.66 ± 2.51
BPH	60	134.99 ± 15.59	20.91 ± 4.39	18.12 ± 3.94	15.44 ± 2.30
*t*		1.604	34.588	1.782	1.915
*p*		0.109	<0.001	0.075	0.056
IIEF-5					
BPH + PI	52	19.34 ± 3.36	10.64 ± 2.23	12.35 ± 1.99	14.47 ± 1.88
BPH	60	19.76 ± 2.98	11.21 ± 2.30	13.16 ± 2.24	14.65 ± 1.96
*t*		3.043	4.309	7.019	1.844
*p*		0.002	<0.001	<0.001	0.065

### Comparison of postoperative adverse events between the two groups

The incidence of postoperative fever, urinary tract irritation, bladder spasm, acute epididymitis, urinary tract infection, urethral stricture, and intraoperative prostate capsule perforation in the BPH + PI group was significantly higher than that in the BPH group (*p* < 0.05). There was no significant difference between the two groups in terms of secondary surgery, blood transfusion, bladder injury, urinary incontinence, and overall postoperative complications (*p* > 0.05). The number of patients with postoperative pathology suggesting prostate adenocarcinoma in the BPH + PI group was higher than that in the BPH group (*p* < 0.05) (Table 4).

**Table 4:** Comparison of postoperative adverse events between the two groups [*n* (%)].

Index	BPH + PI	BPH	*x* ^2^	*p*
Pathology prostate cancer (*n*)	6 (11.54)	1 (1.67)	4.633	0.031
Second surgery (*n*)	2 (3.85)	1 (1.67)	0.508	0.90
Fever > 38.5°C (*n*)	9 (17.31)	2 (3.33)	6.142	0.013
Blood transfusion (*n*)	1 (1.92)	1 (1.67)	0.01	1.00
Bladder damage (*n*)	2 (3.85)	3 (5.00)	0.087	0.768
Prostatic capsule perforation (*n*)	10 (19.23)	3 (5.00)	5.499	0.019
Urinary tract irritation (*n*)	26 (50.00)	15 (25.00)	7.503	0.006
Bladder spasm (*n*)	20 (38.46)	12 (20.00)	4.652	0.031
acute epididymitis (*n*)	6 (11.54)	1 (1.67)	4.633	0.031
Urinary tract infection (*n*)	8 (15.38)	2 (3.33)	4.976	0.026
Transient urinary incontinence^a^ (*n*)	11 (21.15)	12 (20.00)	0.023	0.88
Permanent urinary incontinence^a^ (*n*)	1 (1.92)	2 (3.33)	0.213	1.00
Urethral stricture (*n*)	8 (15.38)	2 (3.33)	4.976	0.026
Clavien–Dindo classification^b^ (*n*)				
I	26 (50.00)	24 (40.00)	1.127	0.288
II	12 (23.08)	12 (20.00)	0.157	0.692
III	6 (11.54)	5 (8.33)	0.323	0.570
IV	2 (3.85)	2 (3.33)	0.021	1.00

^a^ Incontinence is defined as transient incontinence if it improves with treatment within 6 months; if not, it is defined as permanent incontinence.

^b^ If a patient has two or more kinds of complications, the most severe complication was defined as the grade of Clavien–Dindo classification.

## Discussion

BPH and PI are prevalent urinary system disorders among elderly men. It has been observed that over 50% of elderly men experience lower urinary tract symptoms (LUTS) due to BPH, with a significant proportion of these individuals necessitating medical intervention or surgical procedures as they age (1). PI is present in approximately 20% of BPH cases, and even higher rates have been reported. A recent study revealed that among BPH patients with surgical indications, the prevalence of PI was 46%, consistent with findings from previous research (10). However, contrasting results have been reported, with one study indicating a combined BPH and PI incidence of 78.6% (4), which may be related to the difference of pathological collection and diagnostic criteria. Research (3) has demonstrated that the presence of PI can facilitate the advancement of prostatic hyperplasia, resulting in an enlargement of the prostate. In addition, BPH can trigger prostatic histological inflammation, establishing a reciprocal relationship between the two conditions. Nevertheless, our study did not observe a notable discrepancy in prostate volume between patient groups with or without PI, possibly attributable to the limited sample size. Certain individuals with BPH exhibit abnormal PSA levels, which have been identified as a significant risk factor for the progression of BPH. In the cohort of patients with benign prostatic hyperplasia necessitating surgical intervention, the elevation in PSA levels is notably pronounced (5). Additional research (7) has demonstrated that the confluence of benign prostatic hyperplasia and prostatic histological inflammation can exacerbate the deterioration of prostate epithelial cells, leading to an escalation in serum PSA levels. Consistent with these findings, our study revealed a heightened average PSA level in patients with benign prostatic hyperplasia and prostatic histological inflammation. Consequently, a prostate biopsy is frequently recommended for such patients before surgical intervention for benign prostatic hyperplasia. One study (9) indicates that the presence of PI in patients with BPH can complicate surgical procedures and result in poorer outcomes compared to BPH patients without PI. However, this conclusion is based on a retrospective analysis of postoperative pathology. It raises the question of whether preoperative identification of PI in BPH patients can inform decisions regarding the timing of surgery.

This study involved the collection of 112 patients with elevated PSA levels and indications for BPH surgery who underwent biopsies. The patients were divided into two groups based on the presence of pathological results combined with PI, and each group underwent transurethral plasma enucleation of the prostate. The surgical outcomes of the two groups were compared. An analysis of the basic data revealed that BPH patients with PI were more prone to experiencing urinary retention after prostate biopsy. This phenomenon may be attributed to the propensity of prostate tissue to exhibit inflammatory edema after biopsy, which is likely a result of the inherent inflammatory response. These findings align with the conclusions drawn in a comprehensive meta-analysis conducted by Bhanji et al. (11), indicating that patients with prostatitis are at a heightened risk of experiencing adverse events after biopsy based on pathological evidence.

This study demonstrates a significant difference in operation time between patients with BPH with PI compared to those with BPH alone. The prolonged operation time in the BPH + PI group may be attributed to difficulties in identifying the prostate level due to inflammation, leading to challenges in locating the correct capsular surface because of presence of adhesions. Similar findings were found in the study by Ottaiano et al. (12). The average time for transurethral surgery in BPH patients with inflammation was longer than that in patients with simple BPH. Simultaneously, our study revealed a notable correlation between inflammation in patients and heightened intraoperative bleeding, as well as a greater necessity for saline flush during surgery compared to those in the non-inflammatory BPH group. Postoperative laboratory analyses further supported these findings, demonstrating a significantly higher decrease in hemoglobin levels on the first day after surgery in the BPH group with inflammation compared to the non-inflammatory BPH group, consistent with prior research conducted by Romero-Otero et al. (13). They also found that histology prostatic histological inflammation is a risk factor for intraoperative bleeding. In the findings of this study, no statistically significant disparity was observed in the flushing time and flushing volume between the two cohorts of patients postoperatively. This phenomenon may be attributed to the thorough hemostasis achieved during the surgical procedure, rather than being influenced by the presence of inflammation. Nevertheless, extant literature posits that the existence of prostatic histological inflammation may heighten the likelihood of postoperative hemorrhage and extend the duration of postoperative flushing (9).

In the findings of this study, patients with PI exhibited elevated postoperative infection markers compared to patients without PI, such as increased levels of postoperative white blood cells and C-reactive protein, as well as a higher incidence of postoperative fever. Xu et al. (14) conducted an analysis on the risk factors for urinary tract infections after prostatic hyperplasia surgery, revealing that BPH patients with prostatitis faced a significantly heightened risk of postoperative infection and fever, both of which could be effectively mitigated through the administration of antibiotics. This phenomenon may be attributed to the destruction of prostate tissue during surgical procedures and the subsequent rise in pressure due to intraoperative perfusion, leading to the infiltration of inflammatory mediators into the bloodstream (15). It remains to be confirmed through additional clinical studies whether preoperative administration of anti-inflammatory therapy to patients with BPH combined with PI can effectively mitigate the likelihood of postoperative infections.

The findings of this study indicate that the preoperative IPSS and IIEF-5 scores were significantly worse in the BPH + PI group compared to the BPH group. This suggests that in the absence of other significant variables, the presence of prostatic histological inflammation may exacerbate prostate symptoms (16) and impair sexual function (17), ultimately impacting the quality of life of patients. In addition, previous research (18) has suggested that both preoperative and postoperative quality of life scores are inferior in BPH patients with prostatic histological inflammation compared to those without prostatic histological inflammation. In this study, the preoperative quality of life score of the BPH + PI group was found to be inferior to that of the BPH group, although the observed difference was not statistically significant, possibly due to the limited sample size. Two weeks postoperatively, patients with inflammation exhibited poorer scores in IPSS, IIEF-5, PVR, and Qmax compared to patients in the BPH group; however, no significant disparities were observed in these parameters between the two groups at the 1-month follow-up. These findings suggest that the presence of inflammation may impact the postoperative recovery trajectory of patients. As postoperative inflammation subsides, the surgical outcome remains stable. Nevertheless, the increased symptom burden in the immediate postoperative period negatively impacts the patient's quality of life. Within 1 month postoperatively, the quality of life score of patients in the BPH + PI group was significantly lower compared to that of patients in the BPH group. However, as other scores and symptoms improved over time, the overall quality of life gradually increased. These findings align with the study by Arora et al., which suggested that patients with prostatic hyperplasia and inflammation may experience a prolonged recovery period after surgery, albeit without significant differences in final surgical outcomes (19).

The findings of this study suggest that while there is no notable disparity in the overall complication rate between the two patient groups in terms of surgical safety, individuals with PI exhibit a significantly higher incidence of infection-related adverse events compared to those with BPH alone. These events include postoperative lower urinary tract symptoms, epididymitis, urinary bacterial infection, and fever, which align with the results reported by Huang et al. (20) in their study on postoperative inflammation-related complications after BPH surgery, including lower urinary tract symptoms. This study found that prostatic histological inflammation led to adhesion and indistinct layers of the prostate capsule during surgery, resulting in a higher incidence of capsular perforation and increased surgical risk. In addition, patients in the prostatic histological inflammation group had a significantly elevated risk of postoperative urethral stricture. Gür et al. (21) conducted a study indicating that the development of short-term urethral stricture after prostatic hyperplasia surgery is associated with intraoperative urethral injury, extended operation duration, and increased postoperative complications, aligning with our own findings. Furthermore, patients in the BPH + PI group exhibited a higher average pain score 3 days postoperatively compared to those in the BPH group. In addition, Sommer et al. (22) discovered that inflammation may exacerbate neuropathic pain severity. Furthermore, the findings of this study suggest that patients in the BPH + PI group had a higher incidence of postoperative pathology reports indicating prostate cancer compared to those in the BPH group. This highlights the potential for prostatic histological inflammation to contribute to an elevated rate of misinterpretation by pathologists when analyzing biopsy specimens. Consequently, patients diagnosed with benign prostatic histological inflammation based on biopsy pathology reports should undergo more vigilant monitoring and consider undergoing a second biopsy to ensure accurate diagnosis and appropriate management.

The findings of this study partially demonstrate the influence of prostatic histological inflammation on prostate enucleation surgery after prostate biopsy, yet several limitations exist. These include the study's single-center design, which may not be generalizable to other medical facilities. Furthermore, the small sample size and short follow-up duration could potentially impact the study's outcomes. In addition, the study did not account for variations in the degree of prostatic histological inflammation or the time interval between surgery and prostate biopsy. De Nunzio et al. (10) have highlighted the significance of prostatic histological inflammation in the management of symptoms in patients with BPH. Our future research will focus on investigating the treatment of inflammation before surgery and determining the optimal timing for surgery after biopsy in this patient population.

In conclusion, while the presence of prostatic histological inflammation does not significantly impact the overall complications and surgical outcomes in patients undergoing prostate enucleation immediately after biopsy, it may increase the risk of infection-related adverse events and hinder the patient's recovery process. Prolonged postoperative recovery time and increased symptom burden may be experienced by patients with pre-existing prostatic histological inflammation undergoing surgery. Preoperative anti-inflammatory treatment may be considered a potential intervention to mitigate these effects, and additional clinical research is needed to evaluate the efficacy and optimal timing of such interventions.

## Data Availability

The original contributions presented in the study are included in the article/Supplementary Material, further inquiries can be directed to the corresponding author.
